# Childhood mental health and juvenile delinquency: A within-family comparison

**DOI:** 10.1192/j.eurpsy.2021.620

**Published:** 2021-08-13

**Authors:** S. Kim, D.H. Kim

**Affiliations:** 1 Psychiatry, Ajou university school of medicine, Suwon, Korea, Republic of; 2 Economics, Myongji University, seoul, Korea, Republic of

**Keywords:** childhood, mental health, ADHD, delinquency

## Abstract

**Introduction:**

Child metal health is associated with prospective delinquent outcomes. However, this association might be confounded by genetic and other shared factors

**Objectives:**

We aimed to examine the association between the behavioral symptoms of Attention-Deficit/Hyperactivity Disorder, conduct disorder, depression, and oppositional defiant disorder in childhood (age 4-12) and the prospective delinquent outcomes as measured by lifetime illicit drug use, criminal activities, and victimization prior to age 18, using the nationally representative U.S. survey that allowed us to compare siblings in the same mother.

**Methods:**

Aged-adjusted subscales or ADHD, conduct disorder, depression, and ODD were obtained from the mother-reported survey responses. Within-family analyses were performed to control for family-specific unobserved factors as well as child-specific observed factors.

**Results:**

Antisocial scores in childhood were strongly associated with lifetime arrest, probation, and incarceration as well as lifetime illicit drug use in adolescence. ADHD scores are associated with lifetime victimization in physical attack and rape, but not with criminal activities or illicit drug use.

**Conclusions:**

Conduct disorder consistently increases lifetime illicit drug use and criminal activities independently of genetic factors and gender. ADHD is not associated with lifetime illicit drug use or criminal activities, but is associated with lifetime victimization. No significant gender differences are found although anxiety/depression symptoms are often positively associated with delinquent outcomes only among females.

